# The Ethics of Nursing1Introductory lecture to the first course at the Brooks Memorial Hospital Training School for Nurses, delivered October 1, 1901.

**Published:** 1901-11

**Authors:** Geo. E. Blackham

**Affiliations:** Dunkirk, N. Y., President of the Medical and Surgical Staff of the Brooks Memorial Hospital.


### The Ethics of Nursing.[1]

By GEO. E. BLACKHAM, M.D., Dunkirk, N. Y.,
President of the Medical and Surgical Staff of the Brooks Memorial Hospital.

IT GIVES me great pleasure to offer you at the outset of your professional careers, a hearty welcome from the board of directors and the medical and surgical staff. You have chosen a high and honorable calling—one closely allied to that of the Master himself, who, we are told, "went about doing good" and offered us evidence of his divine mission, the fact that through his ministration the blind see, and the deaf hear, and the dead are raised up. While such wonders may not mark your path, yet I hope much suffering may be alleviated and many sick ones be healed through your work.

So, then, the mission of the trained nurse is essentially one of comfort and healing. If you have chosen this path through any unworthy motive, merely as an easier or more genteel way of making a living than working or washing dishes, book-keeping or stenography,—if you have chosen it merely for the money there is in it for you, you have chosen unwisely and would better give it up now. But if you have chosen it because, in addition to providing you with an honorable means of livelihood, it also offers you almost unlimited opportunities for doing good, then you have chosen wisely and I exhort you to continue in the face of all the difficulties and discomforts, and they are many, that will constantly beset your path.

To do your work well you must learn many things and you have a distinct advantage in beginning your studies in a small hospital, where you can receive more individual instruction than if you were members of a large class in a large hospital where the patients are classified into departments and you could see only a single class at a time. Here, too, the conditions approximate more closely to those which will confront you in private practice. Your superintendent, Miss Crysler, will see to it that you have opportunities to learn and practice the details of your profession, and members of the medical and surgical staff will give you instructions by means of lectures and demonstrations, but after all, the extent to which you will profit by these opportunities will depend chiefly upon yourselves.

You must study and work—work and study—deeming nothing too small or unimportant to receive your full attention.

1. Introductory lecture to the first course at the Brooks Memorial Hospital Training School for Nurses, delivered October 1, 1901.

As George Herbert has well said:

> "A servant with this clause
> Makes drudgery divine—
> Who sweeps a room as for Thy laws—
> Makes that and th' action fine."

Nor on the other hand must you deem anything too great or too deep for you to learn all you can about it. Of the mysteries of anatomy and physiology, of materia medica and pharmacy, you must have some knowledge, but in acquiring this you must not neglect the minor matters which are after all more important for a good nurse to know.

It is a far cry from the Sary Gamps and Betsey Prigs who dropped their snuff into the unfortunate patients broth and addled their own already dull brains with gin and water, to the neat, alert, capable and conscientious trained nurse of the twentieth century, whose presence brings hope and confidence and whose touch brings comfort and healing to the sick and afflicted. The difference is by no means entirely due to the fact that the trained nurse knows more of the technicalities of her profession; it is even more due to that fact that she is a different woman; one with a higher conception of the sacredness of her calling; for a nurse may be able to pass the most rigid examination in every technical subject and yet be as totally unfit for the sick room as poor drunken old Sary Gamp ever was, unless she knows and practises the ethics of her profession.

Ethics has been defined as the science of duty and it is even more the art of doing one's duty. If it be true that in a sense "the manners make the man," it is even more true that manners make the woman, so that you may be learned in all the scientific parts of your profession, may have muscles of iron and nerves of steel and yet fall far short of the ideal of the trained nurse, if you do not know and practice ethics, and it is of this that I have been assigned to speak to you tonight.

Ethics may, then, be briefly defined as the science and art of doing one's duty and the ethics of nursing may conveniently be divided into (1) the duty of the nurse to herself; (2) the duty of the nurse to her patients; (3) the duty of the nurse to the family of the patient; (4) the duty of the nurse to the attending physician or surgeon; (5) the duty of the nurse to her alma mater; (6) the duty of the nurse to the community in general.

The first duty of the nurse is to be clean physically, mentally and morally. The surgery of today differs from that of one hundred or even fifty years ago chiefly in the matter of cleanliness. Not that the older surgeons were not clean, as they understood cleanliness, but since the days of Lister, of Pasteur, and of Koch, cleanliness has taken on a new meaning. In the old days the surgeon operated and washed his hands. Now he washes his hands before he operates and not only that but he does it with an elaboration of detail and a thoroughness and perfection of technique that outrivals the ceremonial ablutions of the Mosaic Code. Now every knife and needle must go through the purgatory of flame or of steam; must soak in cleansing solutions of lysol, cresol, phenol or some other ol of the phenol group, till the last infinitesimal microbe, bacterium or coccus is routed. The patient must be bathed and scrubbed with green soap, ether, alcohol, bichloride solution, etc. Dressings must be sterilised and even the water used to wash the wounds or instruments must be freshly boiled. Verily, the cornerstone of modern surgery is the old prescription that the prophet Elisha gave unto Naaman, the Syrian—"wash and be clean," and the mental and the moral asepsis is as important as the physical.

The trained nurse must keep in training. Medical science is advancing at such a pace, that one who would not be left behind must struggle to keep up; study, observe, reflect, keep posted, keep up to date, and that you may have strength of body and clearness of brain to do so, keep well; observe yourself the sanitary laws that it is your duty to administer to others; see that you dress simply and healthfully; that you eat to live and not live to eat; take plenty of fresh air, of sunshine, and of sleep. To do so will not always be easy, for, as yet, too many families seem to regard the trained nurse merely as an additional domestic who, in some mysterious way, has solved the problem of perpetual motion and become gifted with the ability to minister to the patient night and day without intermissions for rest, exercise or refreshment.

The dress of the trained nurse should be scrupulously plain and neat, the uniform of her school when possible, but always fresh and clean. Her voice should be like Annie Laurie's, low and sweet, and her manner modest but assured—pleasant but reserved. She must learn to control her temper and her tongue, and to err, if at all, on the side of silence rather than loquacity. She should respect herself and her office and by so doing cause others to respect them also.

Second.—The duty of a trained nurse to her patient is first of all to see that the orders of the attending physician are carried out with scrupulous exactness and in such a way as shall cause the least possible disturbance and discomfort.

She must learn to anticipate the wants and needs of her patient and to gratify them so far as is consistent with his good without waiting for him to ask, and above all, without bothering him with well meant but irritating inquiries as to "shall I do this?" or "wouldn't you like that?" As I have already said, her voice must be low and sweet and she must learn to avoid that dreadful stage whisper which some people seem to think the proper method of speech for the sick room and than which I know of nothing more rasping to the nerves of the invalid. She must learn to do her work quietly. How many a sick one has been rudely awakened from the dearly bought slumber by the rattling of coals upon the fire, when, had they been tied up in little paper bags before being brought into the sick room, they might have been placed upon the fire so noiselessly as not to disturb the sleeper.

The manner of the nurse should be cheerful though not frivolous. A dismal face upon the nurse is always noticed by the patient, who can notice anything, and many times by those who seem past noticing, and may add sufficient depression to turn the balance the wrong way. Be cheerful, then, for your own sake, as well as that of the patient, and while not lacking in sympathy for the sufferings of your patient, do not share them too freely nor look too dismally into the future. The cheery hopefulness of the nurse has lighted the pathway of many a sufferer out of the valley of the shadow.

Be patient, also. Remember that the brain is sick with the rest of the body and the patient therefore irritable and unreasonable and liable to murmur and complain of your best and wisest efforts for his, or her, comfort and welfare. Bear with him then. Repress the sharp retort that will often rise unbidden to your lips but should never be allowed to pass them.

Third.—To the family of the patient the trained nurse should come as a friend in need; a very present help in time of trouble. She comes to bring trained skill in place of ignorant affection, to replace unreasoning anxiety with cool rational knowledge and to relieve relatives and friends, so far as may be, of the responsibility for and, to a much less degree, the labor of caring for the patient. Her face must be guarded as well as her tongue, for the eye of affection is made keen by apprehension and is quick to detect, or fancy it detects, unfavorable omens in the face of the nurse. It is often difficult for the nurse to strike the happy medium between too great and too little familiarity with the family, between too great and too little willingness to spend and be spent in the service of her patient, and it is better to err on the side of reserve rather than familiarity, and of willingness rather than unwillingness to labor and to watch. See that the family respect your position and your rights, but be not too rigid nor exacting as to what are your rights nor too harsh in your manner of securing them.

Always remember that your position in the sick room is one of honor and trust as well as responsibility, that the door of many a secret closet will necessarily be opened to you, and many a hideous skeleton revealed, but that the confidences of the sick room are as sacred as those of the confessional, and that you must be ever on your guard lest by word, sign or look you might betray any portion of the sacred confidence reposed in you. This task of keeping a serious secret is far less difficult than to maintain the necessary reserve in reference to the little things of the sick room, and yet the little task is often as important. The best way is to say as little as possible about your patient or the case.

Fourth.—To the attending physician the trained nurse comes as a tried and trusted lieutenant, one who can be depended upon to carry out his orders with intelligence and accuracy; who will guard his interests as well as those of the patient, keeping him informed of all that occurs between his visits that may be of value to him in his management of the case, and above all refraining from any criticism by word, look or gesture, which might tend to shake the confidence of the patient or the family in his skill, ability or devotion. It is the physician, not the nurse, who is finally responsible for the treatment of the case. He is presumed to have a wider, deeper, more thorough knowledge of what is wrong and of what should or should not be done to set it right and it is for the nurse to coöperate with him to the best of her ability, not to criticise, much less to disobey or deceive one who carries the dread responsibilities of life and death.

It is best never to discuss, even among yourselves, the respective merits of various physicians whose patients you have nursed.

Fifth.—To your alma mater—the training school of Brooks Memorial Hospital—your first duty is to remember it with affection, to so conduct yourselves as to reflect credit upon it; to remember that it cannot, and does not pretend to, complete your education, but to start you right; to set your feet in a path that leads onward and upwards and whose end you may never hope to reach while life shall last.

Sixth.—To the community at large the trained nurse owes the duty of disseminating, by example at least, some knowledge of the principles of her profession, of the gospel of cleanliness, fresh-air, temperance and cheerfulness, of reliance upon medical science rather than upon the vain and boastful claims of the patent medicine manufacturer with his universal panacea, or the unchristian unscientist whose efforts for the relief of suffering consist in the iteration of the inverted axiom that "disease and death cannot and do not exist."

It is your duty to demonstrate to the world at large that you occupy honorable positions in the ranks of that great army which is fighting disease and death, and incidentally sin, and that you are ready, if need be, to sacrifice comfort, health, yea even life itself, as many a nurse, trained and untrained, has done before you, in your high and holy calling of bringing comfort, consolation and healing to the sick and suffering.

When I think of a trained nurse, I am reminded of the little girl who said that woman was a verb, because a verb was to be, to do, and to suffer. God, in making you women, gave you the capacity for being, doing, and suffering, and we in this training school will earnestly strive to teach you how to be, to do, yes, and if need be to suffer for the best good of your fellow mortal immortals. And I hope that when we assemble here again two years hence we may be able to present each one of you with a certificate of having passed successfully through the prescribed course of study and experience necessary to fit you for your profession and that we may send you forth the advance guard of an army "of honorable women not a few" who shall go out from this school well fitted to battle with disease and death and bring ease and comfort to many a sick room. Not yours nor theirs, the marching host, the glittering blades, the martial music, the pomp and circumstance of war, but the snowy cap or the red cross chevron are no less honorable emblems of valor and conquest than waving banners and golden epaulets—it is more glorious to save than to destroy.

This truth has been most beautifully expressed by that great poet and physician, the genial Autocrat of the Breakfast Table, the late Dr. Oliver Wendell Holmes in his poem of "The Two Armies."

"As Life's unending column pours,
Two marshalled hosts are seen,
Two armies on the trampled shores
That Death flows black between.

One marches to the drum beat's roll,
The wide-mouthed clarion's bray,
And bears upon a crimson scroll—
'Our Glory is to slay.'

One moves in silence by the stream,

With sad, yet watchful eyes,

Calm as the patient planet's gleam,

That walks the clouded skies.

Along its front no sabres shine,

No blood-red pennons wave;

Its banner bears the single line,

'Our duty is to save.'

For those—no death-bed's lingering shade;

At Honor's trumpet call—

With knitted brow and lifted blade

In glory's arms they fall.

For these no flashing falchions bright,

No stirring battle-cry;

The bloodless stabber calls by night—

Each answers—' Here am I."

For those the sculptor's laureled
bust,
The builder's marble piles,
The anthems pealing o'er their dust
Through long cathedral aisles.

For these the blossom sprinkled turf

That floods the lonely graves

When Spring rolls in her sea-green surf

In flowery foaming waves.

Two paths lead upward from below,

And angels wait above,

Who count each burning life-drop's flow,

Each falling tear of love.

Though from the hero's bleeding breast

Her pulses freedom drew,

Though the white lilies on her crest,

Sprang from that scarlet dew,

While valor's haughty champions wait

Till all their scars are shown,

Love walks—unchallenged—through the gate,

To sit beside the throne."

332 DOVE STREET.

